# Sampled-data velocity-free consensus of Multiple Euler-Lagrange systems under irregular communication delays

**DOI:** 10.1371/journal.pone.0333896

**Published:** 2025-10-17

**Authors:** Yilin Wang, Jiahao Dai, Pengfei Zhang

**Affiliations:** 1 School of Automation and Electrical Engineering, Zhejiang University of Science & Technology, Hangzhou, China; 2 Huzhou Key Laboratory of Intelligent Sensing and Optimal Control for Industrial Systems, School of Engineering, Huzhou University, Huzhou, China; Whale Wave Technology Inc, CHINA

## Abstract

This paper addresses the challenging problem of achieving sampled-data, velocity-free consensus for multiple Euler-Lagrange systems under irregular communication delays. While passivity-based control (PBC) is a powerful framework for such systems, existing works fundamentally require continuous feedback from neighbors, as their stability proofs cannot handle the discontinuous right-hand-side dynamics generated by sampled-data and abrupt delays. This limitation renders conventional PBC methods inapplicable in many realistic networked scenarios. This work bridges that theoretical gap by introducing a novel control and analysis method. Our strategy treats the system dynamics over continuous intervals separately from the discrete instants of discontinuity, allowing us to rigorously prove consensus. The control strategy incorporates a virtual system framework to operate without velocity measurements and successfully relaxes the impractical requirement that delays must have finite derivatives. Finally, simulation examples are provided to demonstrate the effectiveness of the proposed consensus algorithm. Index terms: Euler-Lagrange system, Multi-agent system, Sampled-data control.

## 1. Introduction

The consensus problem of multi-agent systems has been a central and widely studied topic for decades. Numerous efforts have been dedicated to solving a vast range of pertinent problems within this field, as consensus remains the most common control objective for multi-agent systems [1–11], from which studies like synchronization and formation control are derived. The practical applications of consensus are diverse, spanning fields such as biology, physics, control systems, and robotics.

A central concern in these studies is the dynamic models of the individual agents, as a theoretical formulation must properly reflect the actual physical system by representing its physical characteristics. The Euler-Lagrange (EL) system is particularly promising because it can describe a wide range of physical systems, including mechanical, electrical, and electromechanical systems. This advantage makes the Euler-Lagrange system an ideal characterization for agent dynamics and has inspired significant research interest in networked Euler-Lagrange systems [1–9, 12–14].

However, a significant challenge arises in many real-world systems: velocity measurements are often unavailable or unreliable [15,16]. This is because velocity measurements may require additional sensors that can introduce noise or increase costs, and commercially available devices are often not equipped with them. Existing control methods for Euler-Lagrange systems often rely on the continuous availability of velocity information, which is impractical in scenarios lacking velocity sensors or with limited communication bandwidth.

Communication is another critical aspect in multi-agent systems. While much of the existing literature assumes continuous communication, modern computer-based networked systems, such as vehicle formations, drone swarms, and satellite constellations, rely exclusively on sampled-data communication, which is more realistic and practical [5,17,18]. Furthermore, communication delay is an unavoidable and common constraint in multi-agent systems, as it is often time-varying in the real world due to multiple influencing factors. Therefore, these delays must be carefully considered when designing control algorithms.

Our control strategy employs a passivity-based control (PBC) framework [19–22] to achieve consensus. The PBC methodology leverages energy shaping and damping injection principles, where the energies of the systems and the virtual energies of the controllers (virtual systems) are combined to form a suitable Lyapunov function, and damping is added to the controllers ensure asymptotic stability. A key advantage of the virtual system is its ability to inject necessary damping without relying on velocity measurements of the EL systems [19,22], which is crucial as many commercially available devices lack velocity sensors or are prone to noise. This approach is also inherently robust to interconnecting time-varying delays [23].

The challenge of unavailable velocity measurements is specifically addressed by incorporating a virtual system framework. This approach, as introduced in [23], pairs each EL system with a virtual system (dynamic controller). This allows the EL systems to achieve consensus without directly using the generalized velocities of the agents, which are often difficult to obtain in practice.

While prior works have successfully utilized this virtual system framework for EL systems with communication delays, a notable limitation was the requirement that delays must have finite derivatives. This assumption proves impractical in real-world scenarios where delays can be irregular, i.e., discontinuous. This work resolves this issue through a novel analytical approach that handles the discontinuous system dynamics. Our proof strategy is twofold:

1. First, we prove that consensus is achieved on the continuous intervals of the system’s trajectory as time approaches infinity. This result is established independently of the system’s behavior at the moments of discontinuity.
2. Next, we demonstrate the continuity of the generalized and virtual states at the discrete time instants where the dynamics are discontinuous.

By combining the proof of consensus on continuous intervals with the proof of state continuity at the discontinuous points, we can rigorously establish the final consensus result for the entire system trajectory. This method allows us to relax the requirement for finite delay derivatives, enabling the application of the PBC method in situations with irregular delays, which includes sampled-data communication environments where information is piece-wise constant and updates abruptly at sampling instants. This approach also gives a definitive minimum value of the damping coefficient which can be used to guide the controller design. The contributions of this work can be summarized as follows:

1. Extends the passivity-based control (PBC) framework to systems with irregular, discontinuous communication delays and sampled-data communication by removing the impractical requirement that delays must have finite derivatives.
2. Establishes a definitive, sufficient condition for the controller’s damping gains that guarantees consensus and provides a clear guideline for controller design.
3. Employs an analytical technique that proves consensus by separately considering the system’s behavior on continuous intervals and at the discrete instants of discontinuity, an approach with potential application to other discontinuous control problems like event-triggered control.

The rest of the paper is organized as follows. Section II provides preliminaries on the problem investigated. Section III introduces PBC and virtual system scheme for EL systems and provides the analysis. Finally, numerical examples are given in Section IV to verify the theoretical results.

## 2. Preliminaries

Consider a total of N networked Euler-Lagrange systems that are fully actuated with the following dynamics:


$$\begin{array}{c} M_{i}\left(q_{i}\right){\ddot{q}}_{i}+C_{i}\left(q_{i},{\dot{q}}_{i}\right){\dot{q}}_{i}+g_{i}\left(q_{i}\right)=\Gamma_{i},\;i=\text{1},\;\text{2},\;\ldots ,\;N \end{array}$$ (1)

where $q_{i}={\left[q_{i\text{1}},\;q_{i\text{2}},\;\ldots ,\;q_{in}\right]}^{T}\in R^{n}$ is the generalized position, $M_{i}\left(q_{i}\right)=M_{i}^{T}\left(q_{i}\right)\in R^{n\times n\;}$ is the inertia matrix, $C_{i}\left(q_{i},{\dot{q}}_{i}\right)\in R^{n\times n\;}$ is the Coriolis and centrifugal matrix, $g_{i}\left(q_{i}\right)\in R^{n}$ is the gravitational torque, $\tau_{i}=\in R^{n}$ is the control input, and the following general assumptions hold for all the Euler-Lagrange system (1):

1) There exist positive-definite parameters $k_{c}$ and $k_{d}$ such that $\text{0}<k_{c}I_{n}\leq M_{i}\left(q_{i}\right)\leq k_{d}I_{n}$.
2) ${\dot{M}}_{i}\left(q_{i}\right)-\text{2}C_{i}\left(q_{i},{\dot{q}}_{i}\right)$ is skew-symmetric, i.e., for any $r\in R^{n}$, $r^{T}\left({\dot{M}}_{i}\left(q_{i}\right)-\text{2}C_{i}\left(q_{i},{\dot{q}}_{i}\right)\right)r=\text{0}$.

Communication links among the agents are described by a weighted undirected graph G = {V, E,A}, where V = {1,2,...,N} is the set of all nodes, E= V×V is the set of edges, and $A\;=\;{\left(a_{ij}\right)}_{N\times N}$ is the weighted adjacency matrix. {j,i}∈E indicates that agent i receives information from agent j, i.e., j is a neighbor of i. The set of all i’s neighbors is denoted $N_{i}$ An ordered sequence of distinct edges of G in the form $\left\{\left(j,l_{\text{1}}\right),\left(l_{\text{1}},l_{\text{2}}\right),...,\left(l_{k},i\right)\right\}$ is called a directed path from j to i. A directed graph G contains a spanning tree if there exists at least one node that has a directed path to all the other nodes, and that one node is called the root of the spanning tree. $a_{ij}>\;\text{0}$ if and only if (j,i)∈E, otherwise $a_{ij}=\text{0}$. Assume that there is no self-loop, i.e., $a_{ii}=\text{0}$, i ∈V. Let $\Delta_{i}=\sum_{j=\text{1}}^{N}a_{ij}=\sum_{{j\in N}_{i}}a_{ij}$, $\Delta =diag\left(\Delta_{\text{1}},\ldots ,\Delta_{N}\right)$. The Laplacian matrix is $L={\left(l_{ij}\right)}_{N\times N}=\Delta -A$.

Each agent samples at its own sampling instants, e.g., $t_{\text{0}}^{i},\;t_{\text{1}}^{i},\;\ldots ,\;t_{k}^{i},\;\ldots$ for agent i, and $t_{\text{0}}^{i}=\text{0}$. Each agent could have different sampling instants and different sampling intervals. The only universal constraint is that all the sampling intervals are upper-bounded by h, i.e., $t_{k+\text{1}}^{i}-t_{k}^{i}\leq h$ ∀ i∈V, k≥0.

The time it takes for the sampled state $x_{j}\left(t_{k}^{j}\right)$ to reach agent i is denoted by $\tau_{ij}\left(t_{k}^{j}\right)$, i.e., the communication delays. The communication delays are only known to be upper-bounded by T. We adopt the common assumption that the sequence of data packets on one communication channel is preserved. Since the communication is sampled, no assumption on differentiability or even continuity is needed.

Notations: Let $Diag\left\{A_{\text{1}},\;A_{\text{2}},\;\ldots ,\;A_{n}\right\}$ be the block diagonal matrix whose i’th diagonal block is $A_{i}$. A⊗B denotes the Kronecker product of matrices A and B. For a vector function $f:[\text{0},\;\infty )\to R^{n},\;t\mapsto f(t)$, ${\left|f(t)\right|}_{p}={\left(\sum_{i}^{n}{\left|f_{i}(t)\right|}^{p}\right)}^{\frac{\text{1}}{p}}$ denotes its p-norm, and ${\left\|f(t)\right\|}_{p}={\left(\int_{\text{0}}^{\infty }{\left|f(t)\right|}^{p}dt\right)}^{\frac{\text{1}}{p}}$ its $L_{p}$ norm, and ${\left\|f(t)\right\|}_{\infty }=\underset{t>\text{0}}{\text{sup}}{\left|f(t)\right|}_{\infty }$ its $L_{\infty }$ norm. We call $f(t)\in L_{p}$ if ${\left\|f(t)\right\|}_{p}<\infty$. Throughout this work, the functional dependence on time t will be implicitly assumed for all dynamic variables. For example, the variable x represents the function x(t).

### 2.1. Control objectives

Consider the N networked Euler-Lagrange systems in (1) with a connected undirected communication graph, assume that velocity measurements are unavailable. The communication delays $\tau_{ij}\left(t_{k}^{j}\right)$ are time-varying, and different on different edges (i,j). The goal is to construct a decentralized consensus algorithm, i.e., control torque $\Gamma_{i}$ that solves the consensus problems


$$\underset{t\to \infty }{\text{lim}}\left(q_{i}(\text{t})-q_{j}(t)\right)=\text{0},\;\underset{t\to \infty }{\text{lim}}{\dot{q}}_{i}(\text{t})=\text{0}$$

and derive the conesensus conditions.

### 2.2. Useful lemma and corollary

Lemma 1 (Barbalat’s lemma): Assume that f is a function of time only, If f(t) has a finite limit as t→∞ and if $\dot{f}$ is uniformly continuous (or $\ddot{f}$ is bounded), then $\dot{f}(t)\to \text{0}$ as t→∞.

Corollay 1 [24]: If $f\in L_{\text{2}}$ and $\dot{f}\in L_{\infty }$, then f(t)→0 as t→∞.

## 3. Main results

Since the velocity measurements of the EL systems is not available, damping cannot be directly applied to ensure asymptotic stability. We adopt the PBC method of [23] that is equivalent to appending a virtual system to each of the EL systems and inject the damping to the virtual systems instead of the actual EL systems. Denote the virtual systems’ position by $x_{i},\;i=\text{1},\;\ldots ,\;N$ and velocities by $v_{i},\;i=\text{1},\;\ldots ,\;N$

The virtual system needs to be passive to dissipate energy, so a power-preserving interconnection is needed between the EL system and the virtual system. We choose a virtual spring to connect the EL system and the virtual system, and the potential energy is


$$\begin{array}{c} U_{i}=\frac{\text{1}}{\text{2}}k_{i}{\left(q_{i}-x_{i}\right)}^{\text{2}} \end{array}$$ (2)

And to preserve energy, on the EL system side, the control torque $\Gamma_{i}$ should satisfy that


$$\begin{array}{c} M_{i}\left(q_{i}\right){\ddot{q}}_{i}+C_{i}\left(q_{i},{\dot{q}}_{i}\right){\dot{q}}_{i}=-\frac{\partial U_{i}}{\partial q_{i}}=-k_{i}\left(q_{i}-x_{i}\right) \end{array}$$ (3)

So it is obvious that


$$\begin{array}{c} \Gamma_{i}=g_{i}\left(q_{i}\right)-k_{i}\left(q_{i}-x_{i}\right),\;i=\text{1},\text{2},\ldots ,N \end{array}$$ (4)

And on the virtual system side, we design that its virtual mass is 1, so when connected with only the EL system, its acceleration is


$$\begin{array}{c} a_{i}=-\frac{\partial U_{i}}{\partial q_{i}}=k_{i}\left(q_{i}-x_{i}\right) \end{array}$$ (5)

Adding the damping $-d_{i}v_{i}$ and the interaction with other systems, the acceleration becomes


$$\begin{array}{c} {\dot{v}}_{i}=-d_{i}v_{i}+k_{i}\left(q_{i}-x_{i}\right)+u_{i} \end{array}$$ (6)

Where $u_{i}$ is the interaction (virtual force) exerted by the neighboring agents. Each of the bidirectional connection between two agents i and j are designed as a virtual spring with coefficient $a_{ij}$, whose potential energy is


$$\begin{array}{c} U_{ij}=\frac{\text{1}}{\text{2}}a_{ij}{\left(x_{i}-x_{j}\right)}^{\text{2}} \end{array}$$ (7)

And therefore, $u_{i}$ is the acceleration caused by all the virtual springs acting on the i’th virtual system:


$$u_{i}=-\sum_{N_{i}}\frac{\partial U_{ij}}{\partial x_{i}}=-\sum_{N_{i}}a_{ij}\left(x_{i}-x_{j}\right)$$ (8)

where $N_{i}$ is the set of agent i’s neighboring agents. However, this expression for $u_{i}$ is only valid for ideal, continuous communication without delays. This work considers both sampled-data communication and varying communication delays.

The time it takes for the sampled state $x_{j}\left(t_{k}^{j}\right)$ to reach agent i is denoted by $\tau_{ij}\left(t_{k}^{j}\right)$, i.e., the communication delays. The neighbor’s data $x_{j}$ is subject to sampling and delay before it is used in $u_{i}$, and we denote this received data from the neighbor j by $x_{ij}(t)$, that is the latest sampled state of agent j that is received by agent i. By this definition, we have


$$\begin{array}{c} x_{ij}(t)≝x_{j}\left(t_{k_{ij}(t)}^{j}\right) \end{array}\;$$ (9)

where


$$\begin{array}{c} k_{ij}(t)=\text{max}\left\{k|t_{k}^{j}+\tau_{ij}\left(t_{k}^{j}\right)\leq t\right\} \end{array}$$ (10)

Therefore, the interaction between the i’th virtual system with its neighbors can be written as


$$u_{i}=-\sum_{N_{i}}a_{ij}\left(x_{i}-x_{ij}(t)\right)$$ (11)

Obviously $x_{ij}(t)$ is piecewise constant. It can be seen that $x_{ij}(t)$ is a delayed $x_{j}(t)$, and the time delay has an upper bound:

Lemma 3: $t-t_{k_{ij}(t)}^{j}<T+h$.

Proof: By the definition of $t_{k_{ij}(t)}^{j}$, $t_{k_{ij}(t)}^{j}+\tau_{ij}\left(t_{k_{ij}(t)}^{j}\right)\leq t<t_{k_{ij}(t)+\text{1}}^{j}+\tau_{ij}\left(t_{k_{ij}(t)+\text{1}}^{j}\right)$, then


$$\tau_{ij}\left(t_{k_{ij}(t)}^{j}\right)\leq t-t_{k_{ij}(t)}^{j}<t_{k_{ij}(t)+\text{1}}^{j}+\tau_{ij}\left(t_{k_{ij}(t)+\text{1}}^{j}\right)-t_{k_{ij}(t)}^{j}$$

By definition, $\tau_{ij}\left(t_{k_{ij}(t)+\text{1}}^{j}\right)\leq T$ and $t_{k_{ij}(t)+\text{1}}^{j}-t_{k_{ij}(t)}^{j}\leq h$, therefore, it can be established that


$$\begin{array}{c} t-t_{k_{ij}(t)}^{j}<T+h \end{array}$$

Now, we see the communicaiton delays are upper-bounded by T+h. For future convenience, let


$$\begin{array}{c} T_{ij}(t)=t-t_{k_{ij}(t)}^{j}<T+h \end{array}$$ (12)

And $u_{i}$ can be written as


$$u_{i}=-\sum_{N_{i}}a_{ij}\left(x_{i}-x_{j}\left(t-T_{ij}(t)\right)\right)=-\sum_{N_{i}}a_{ij}\left[\left(x_{i}-x_{j}\right)-\left(x_{j}-x_{j}\left(t-T_{ij}(t)\right)\right)\right]$$ (13)

Therefore, the dynamics of an EL system and its associated virtual system can be written as


$$\begin{array}{c} M_{i}\left(q_{i}\right){\ddot{q}}_{i}+C_{i}\left(q_{i},{\dot{q}}_{i}\right){\dot{q}}_{i}=-k_{i}\left(q_{i}-x_{i}\right) \end{array}$$ (14)

$${\dot{v}}_{i}=-d_{i}v_{i}+k_{i}\left(q_{i}-x_{i}\right)-\sum_{N_{i}}a_{ij}\left[\left(x_{i}-x_{j}\right)-\left(x_{j}-x_{j}\left(t-T_{ij}(t)\right)\right)\right]$$ (15)

Theorem 1: Consider the network of EL-agents (1) with an undirected connected interconnection graph, the dynamics (13–14) solves the consensus problem provided that the gains are set as


$$d_{i}>(T+h)\Delta_{i},\;i=\text{1},\ldots ,N$$ (16)

Proof: Construct a Lyapunov function for the whole system based on the (virtual) mechanical energy stored in the systems, including the kinetic energy and potential energy. Note that ${\dot{x}}_{i}=v_{i}$.


$$V_{\text{1}}=\frac{\text{1}}{\text{2}}\sum_{i=\text{1}}^{N}\left({\dot{q}}_{i}^{T}M_{i}{\dot{q}}_{i}+U_{i}+v_{i}^{T}v_{i}+\frac{\text{1}}{\text{2}}\sum_{N_{i}}U_{ij}\right)$$ (17)

$U_{ij}$ is the virtual potential energy stored in the virtual spring between virtual systems i and j. Since the graph G is undirected, $U_{ij}$ and $U_{ji}$ are both summed once in (17) while they point to the same potential energy, in other words, the same potential energy is summed twice in the summation. Therefore, the $U_{ij}$ terms has an additional coefficient $\frac{\text{1}}{\text{2}}$ in (17). The derivative of V is:


$${\dot{V}}_{\text{1}}=\frac{\text{1}}{\text{2}}\sum_{i=\text{1}}^{N}\left({\ddot{q}}_{i}^{T}M_{i}{\dot{q}}_{i}+{\dot{q}}_{i}^{T}M_{i}{\ddot{q}}_{i}+{\dot{q}}_{i}^{T}{\dot{M}}_{i}{\dot{q}}_{i}+\frac{\partial U_{i}}{\partial x_{i}}{\dot{x}}_{i}+\frac{\partial U_{i}}{\partial q_{i}}{\dot{q}}_{i}+{\dot{v}}_{i}^{T}v_{i}+v_{i}^{T}{\dot{v}}_{i}+\frac{\text{1}}{\text{2}}\sum_{N_{i}}\left(\frac{\partial U_{ij}}{\partial x_{i}}{\dot{x}}_{i}+\frac{\partial U_{ij}}{\partial x_{j}}{\dot{x}}_{j}\right)\right)$$

Incorporating (10,11),


V˙1=∑i=1N(∑Niq˙iT(−ki(qi−xi)−Ci(qi,q˙i)q˙i)+12q˙iTM˙iq˙i+ki(xi−qi)T(x˙i−q˙i)+viT(−divi+ki(qi−xi)−∑Niaij((xi−xj)−(xj−xj(t−Tij(t)))))+ 12∑Niaij(xi−xj)T(x˙i−x˙j))

Use the fact that ${\dot{M}}_{i}\left(q_{i}\right)-\text{2}C_{i}\left(q_{i},{\dot{q}}_{i}\right)$ is skew-symmetric,


V˙1=∑i=1N(∑Ni−kiq˙iT(qi−xi)+ki(xi−qi)(x˙i−q˙i)+viT(−divi+ki(qi−xi)−∑Niaij((xi−xj)−(xj−xj(t−Tij(t)))))+ 12∑Niaij(xi−xj)T(x˙i−x˙j))

And the $-k_{i}{\dot{q}}_{i}^{T}\left(q_{i}-x_{i}\right)+k_{i}\left(x_{i}-q_{i}\mathrm{\backslash rightleft}({\dot{x}}_{i}-{\dot{q}}_{i}\right)+k_{i}v_{i}^{T}\left(q_{i}-x_{i}\right)$ term, i.e., equal to 0. The above equation can be simplified to


$${\dot{V}}_{\text{1}}=\sum_{i=\text{1}}^{N}\left(+v_{i}^{T}\left(-d_{i}v_{i}-\sum_{N_{i}}a_{ij}\;\left(\left(x_{i}-x_{j}\right)-\left(x_{j}-x_{j}\left(t-T_{ij}(t)\right)\right)\right)\;\right)+\frac{\text{1}}{\text{2}}\sum_{N_{i}}a_{ij}{\left(x_{i}-x_{j}\right)}^{T}\left({\dot{x}}_{i}-{\dot{x}}_{j}\right)\right)$$

$$=\sum_{i=\text{1}}^{N}\left(+v_{i}^{T}\left(-d_{i}v_{i}-\sum_{N_{i}}a_{ij}\;\left(\left(x_{i}-x_{j}\right)-\left(x_{j}-x_{j}\left(t-T_{ij}(t)\right)\right)\right)\;\right)+\frac{\text{1}}{\text{2}}\sum_{N_{i}}a_{ij}{\left(x_{i}-x_{j}\right)}^{T}\left(v_{i}-v_{j}\right)\right)$$

Since the communication graph G is undirected,


$$\begin{array}{cc} & \sum_{i=\text{1}}^{N}\sum_{N_{i}}a_{ij}{\left(x_{i}-x_{j}\right)}^{T}\left(v_{i}-v_{j}\right)=\sum_{i=\text{1}}^{N}{\left(v_{i}-v_{j}\right)}^{T}\sum_{N_{i}}a_{ij}\left(x_{i}-x_{j}\right)=\sum_{i=\text{1}}^{N}{v_{i}}^{T}\sum_{N_{i}}a_{ij}\left(x_{i}-x_{j}\right)+\sum_{i=\text{1}}^{N}{v_{j}}^{T}\sum_{N_{i}}a_{ij}\left(x_{j}-x_{i}\right)\\ & =\sum_{i=\text{1}}^{N}{v_{i}}^{T}\sum_{N_{i}}a_{ij}\left(x_{i}-x_{j}\right)+\sum_{j=\text{1}}^{N}{v_{j}}^{T}\sum_{N_{j}}a_{ij}\left(x_{j}-x_{i}\right)=\text{2}\sum_{i=\text{1}}^{N}{v_{i}}^{T}\sum_{N_{i}}a_{ij}\left(x_{i}-x_{j}\right) \end{array}$$

The above derivation leverages the summation across all the entries of the symmetric adjacency matrix A. Substituting the above into $\dot{V}$,


$$\begin{array}{cc} {\dot{V}}_{\text{1}} & =\sum_{i=\text{1}}^{N}\left(v_{i}^{T}\left(-d_{i}v_{i}-\sum_{N_{i}}a_{ij}\left(\left(x_{i}-x_{j}\right)-\left(x_{j}-x_{j}\left(t-T_{ij}(t)\right)\right)\right)\right)+{v_{i}}^{T}\sum_{N_{i}}a_{ij}\left(x_{i}-x_{j}\right)\right)\\ & =\sum_{i=\text{1}}^{N}\left(-d_{i}v_{i}^{T}v_{i}+v_{i}^{T}\sum_{N_{i}}a_{ij}\left(x_{j}-x_{j}\left(t-T_{ij}(t)\right)\right)\right)=\sum_{i=\text{1}}^{N}\left(-d_{i}v_{i}+v_{i}^{T}\sum_{N_{i}}a_{ij}\int_{t-\tau_{ij}}^{t}v_{j}(s)ds\right)\\ & =\sum_{i=\text{1}}^{N}\left(-d_{i}v_{i}^{T}v_{i}+\sum_{N_{i}}a_{ij}v_{i}^{T}\int_{t-\tau_{ij}}^{t}v_{j}(s)ds\right)\leq \sum_{i=\text{1}}^{N}\left(-d_{i}v_{i}^{T}v_{i}+\sum_{N_{i}}a_{ij}\left(\frac{\alpha_{i}}{\text{2}}v_{i}^{T}v_{i}+\frac{\text{1}}{\text{2}\alpha_{i}}{\left(\int_{t-\tau_{ij}}^{t}v_{j}(s)ds\right)}^{\text{2}}\right)\right) \end{array}$$

Where $\alpha_{i}$ is an arbitrary positive real number. We employ the Lyapunov-Krasovskii functional


$$V_{\text{2}}=\sum_{i=\text{1}}^{N}\sum_{j\in N_{i}}a_{ij}\eta_{i}\int_{-T-h}^{\text{0}}\int_{t+s}^{t}v_{j}^{T}(\sigma )v_{j}(\sigma )d\sigma ds$$ (18)

Its time derivative is


$$\begin{array}{cc} {\dot{V}}_{\text{2}} & =\sum_{i=\text{1}}^{N}\sum_{j\in N_{i}}a_{ij}\eta_{i}\int_{-T-h}^{\text{0}}\frac{d\int_{t+s}^{t}v_{j}^{T}(\sigma )v_{j}(\sigma )d\sigma }{dt}ds\\ & =\sum_{i=\text{1}}^{N}\sum_{j\in N_{i}}a_{ij}\eta_{i}\int_{-T-h}^{\text{0}}\left(v_{j}^{T}(t)v_{j}(t)-v_{j}^{T}(t+s)v_{j}(t+s)\right)ds\\ & =\sum_{i=\text{1}}^{N}\sum_{j\in N_{i}}a_{ij}\eta_{i}\left((T+h)v_{j}^{T}(t)v_{j}(t)-\int_{-T-h}^{\text{0}}v_{j}^{T}(t+s)v_{j}(t+s)ds\right)\\ & =\sum_{i=\text{1}}^{N}\sum_{j\in N_{i}}a_{ij}\eta_{i}\left((T+h)v_{j}^{T}(t)v_{j}(t)-\int_{t-T-h}^{t}v_{j}^{T}(s)v_{j}(s)ds\right) \end{array}$$

By the Cauchy-Schwarz inequality, we have


$$-\int_{t-T-h}^{t}v_{j}^{T}(s)v_{j}(s)ds\leq -\frac{\text{1}}{T+h}{\left(\int_{t-T-h}^{t}v_{j}(s)ds\right)}^{\text{2}}$$

Therefore,


$${\dot{V}}_{\text{2}}\leq \sum_{i=\text{1}}^{N}\sum_{j\in N_{i}}a_{ij}\eta_{i}\left((T+h)v_{j}^{T}(t)v_{j}(t)-\frac{\text{1}}{T+h}{\left(\int_{t-T-h}^{t}v_{j}(s)ds\right)}^{\text{2}}\right)$$

Denote $V=V_{\text{1}}+V_{\text{2}}$, then


$$\dot{V}\leq \sum_{i=\text{1}}^{N}\left(-d_{i}v_{i}^{T}v_{i}+\sum_{N_{i}}a_{ij}\left(\frac{\alpha_{i}}{\text{2}}v_{i}^{T}v_{i}+\frac{\text{1}}{\text{2}\alpha_{i}}{\left(\;\int_{t-\tau_{ij}}^{t}v_{j}(s)ds\;\right)}^{\text{2}}+\eta_{i}(T+h)v_{j}^{T}v_{j}-\frac{\eta_{i}}{T+h}{\left(\int_{t-T-h}^{t}v_{j}(s)ds\right)}^{\text{2}}\right)\right)$$

Choosing $\eta_{i}$ such that


$$\frac{\text{1}}{\text{2}\alpha_{i}}-\frac{\eta_{i}}{T+h}=\text{0},\;\eta_{i}=\frac{T+h}{\text{2}\alpha_{i}}$$

And using the fact that


$${\left(\;\int_{t-\tau_{ij}}^{t}v_{j}(s)ds\;\right)}^{\text{2}}\leq {\left(\int_{t-T-h}^{t}v_{j}(s)ds\right)}^{\text{2}}$$

${\dot{V}}_{\text{1}}+{\dot{V}}_{\text{2}}$ can be further bounded by


$$\dot{V}\leq \sum_{i=\text{1}}^{N}\left(-d_{i}v_{i}^{T}v_{i}+\sum_{N_{i}}a_{ij}\left(\frac{\alpha_{i}}{\text{2}}v_{i}^{T}v_{i}+\eta_{i}(T+h)v_{j}^{T}v_{j}\right)\right)=\sum_{i=\text{1}}^{N}\left(-d_{i}v_{i}^{T}v_{i}+\sum_{N_{i}}a_{ij}\left(\frac{\alpha_{i}}{\text{2}}v_{i}^{T}v_{i}+\frac{(T+h{)}^{\text{2}}}{\text{2}\alpha_{i}}v_{j}^{T}v_{j}\right)\right)$$

Since $\alpha_{i}$ is arbitrary, choose $\alpha_{i}=\alpha$ for i=1,…,N. Then, using the fact that the communication graph is undirected, $a_{ij}=a_{ji}$, and due to the summation across all (i,j), $v_{j}^{T}v_{j}$ can be replaced with $v_{i}^{T}v_{i}$, so


$$\dot{V}\leq \sum_{i=\text{1}}^{N}\left(-d_{i}v_{i}^{T}v_{i}+\sum_{N_{i}}a_{ij}\left(\frac{\alpha_{i}}{\text{2}}v_{i}^{T}v_{i}+\frac{(T+h{)}^{\text{2}}}{\text{2}\alpha_{i}}v_{i}^{T}v_{i}\right)\right)$$ (19)

Choose $\alpha_{i}=T+h$ to minimize the right-hand-side of the above inequality, and it becomes


$$\dot{V}\leq \sum_{i=\text{1}}^{N}\left(-d_{i}v_{i}^{T}v_{i}+\sum_{N_{i}}a_{ij}(T+h)v_{i}^{T}v_{i}\right)=\sum_{i=\text{1}}^{N}\left(-d_{i}+(T+h)\sum_{N_{i}}a_{ij}\right)v_{i}^{T}v_{i}=\sum_{i=\text{1}}^{N}\left(-d_{i}+(T+h)\Delta_{i}\right)v_{i}^{T}v_{i}\;$$ (20)

Combined with (16), it can be seen that $\dot{V}\leq -\rho v_{i}^{T}v_{i}$ where ρ is a positive scalar dependent on the selection of $d_{i}$. Integrating it from 0 to t,


$$V(t)-V(\text{0})\leq -\rho \int_{\text{0}}^{t}v_{i}^{T}(s)v_{i}(s)ds$$

$$\int_{\text{0}}^{t}v_{i}^{T}(s)v_{i}(s)ds\leq \frac{\text{1}}{\rho }\left(V(\text{0})-V(t)\right)\leq \frac{\text{1}}{\rho }V(\text{0})$$

and this ensures that $v_{i}\in L_{\text{2}}$ and $V\in L_{\infty }$, then, ${\dot{q}}_{i}$, $v_{i}$, $\left|q_{i}-x_{i}\right|$, $\left|x_{i}-x_{j}\right|$ are all bounded ($L_{\infty }$) since V(t) is radially unbounded with respect to them. Then it can be obtained from (14) and (15) that ${\ddot{q}}_{i}\in L_{\infty }$. And it can be inferred that


$$\begin{array}{c} \left|x_{i}-x_{j}\left(t-T_{ij}(t)\right)\right|\leq \left|x_{i}-x_{j}\right|+\int_{t-T}^{t}\left|v_{j}(s)\right|ds\;\in L_{\infty } \end{array}$$ (21)

Then, it follows from (15) that ${\dot{v}}_{i}\in L_{\infty }$, and Corollay 1 gives $\underset{t\to \infty }{\text{lim}}v_{i}(t)=\text{0}$.

Now, consider the intervals where ${\dot{v}}_{i}$ is continuous, i.e., ${\dot{T}}_{ij}(t)$ is continuous for all $j\in N_{i}$. Differentiating (15) yields


$${\ddot{v}}_{i}=-d_{i}{\dot{v}}_{i}+k\left({\dot{q}}_{i}-v_{i}\right)-\sum_{N_{i}}a_{ij}\left(v_{i}-\left(\text{1}-{\dot{T}}_{ij}(t)\right)v_{j}\left(t-T_{ij}(t)\right)\right)$$ (22)

The boundedness of ${\dot{q}}_{i}$, $v_{i}$, ${\dot{v}}_{i}$ and ${\dot{T}}_{ij}$ ensures that ${\ddot{v}}_{i}\in L_{\infty }$. Then, Barbalat’s lemma leads to $\underset{t\to \infty }{\text{lim}}{\dot{v}}_{i}(t)=\text{0}$.

Differentiating the above equation again yields


$$\frac{d}{dt}{\ddot{v}}_{i}=-d_{i}{\ddot{v}}_{i}+k\left({\ddot{q}}_{i}-{\dot{v}}_{i}\right)-\sum_{N_{i}}a_{ij}\left({\dot{v}}_{i}+{\ddot{T}}_{ij}(t)v_{j}\left(t-T_{ij}(t)\right)-{\left(\text{1}-{\dot{T}}_{ij}(t)\right)}^{\text{2}}v_{j}\left(t-T_{ij}(t)\right)\right)$$ (23)

By the boundedness of ${\ddot{q}}_{i}$, ${\dot{v}}_{i}$, ${\ddot{v}}_{i}$, ${\dot{T}}_{ij}$ and ${\ddot{T}}_{ij}$, $\frac{d}{dt}{\ddot{v}}_{i}\in L_{\infty }$. Apply Barbalat’s lemma again, and we get $\underset{t\to \infty }{\text{lim}}{\ddot{v}}_{i}(t)=\text{0}$. Consequently, $\underset{t\to \infty }{\text{lim}}{\dot{q}}_{i}(t)=\text{0}$, and (14) gives ${\ddot{q}}_{i}\in L_{\infty }$. Then, differentiating both sides of (14) gives


$$\begin{array}{c} {\dot{M}}_{i}\left(q_{i}\right){\ddot{q}}_{i}+M_{i}\left(q_{i}\right){\dddot{q}}_{i}+\frac{\partial C_{i}\left(q_{i},{\dot{q}}_{i}\right)}{\partial q_{i}}{\dot{q}}_{i}{\dot{q}}_{i}+\frac{\partial C_{i}\left(q_{i},{\dot{q}}_{i}\right)}{\partial {\dot{q}}_{i}}{\ddot{q}}_{i}{\dot{q}}_{i}+C_{i}\left(q_{i},{\dot{q}}_{i}\right){\ddot{q}}_{i}=-k_{i}\left({\dot{q}}_{i}-{\dot{x}}_{i}\right)\\ {\dddot{q}}_{i}=M_{i}^{-\text{1}}\left(q_{i}\right)\left(-k_{i}\left({\dot{q}}_{i}-{\dot{x}}_{i}\right)-{\dot{M}}_{i}\left(q_{i}\right){\ddot{q}}_{i}-\frac{\partial C_{i}\left(q_{i},{\dot{q}}_{i}\right)}{\partial q_{i}}{\dot{q}}_{i}{\dot{q}}_{i}-\frac{\partial C_{i}\left(q_{i},{\dot{q}}_{i}\right)}{\partial {\dot{q}}_{i}}{\ddot{q}}_{i}{\dot{q}}_{i}-C_{i}\left(q_{i},{\dot{q}}_{i}\right){\ddot{q}}_{i}\right) \end{array}$$ (24)

The boundedness of ${\dot{x}}_{i}$, ${\dot{q}}_{i}$ and ${\ddot{q}}_{i}$ implies ${\dddot{q}}_{i}\in L_{\infty }$. Using Barbalat’s lemma again yields $\underset{t\to \infty }{\text{lim}}{\ddot{q}}_{i}(t)=\text{0}$, substituting this into (14), it can be derived that


$$\underset{t\to \infty }{\text{lim}}\left(q_{i}(t)-x_{i}(t)\right)=\text{0}$$

Substituting this into (15) and get $\underset{t\to \infty }{\text{lim}}u_{i}(t)=\text{0}$. The fact that $\underset{t\to \infty }{\text{lim}}v_{i}(t)=\text{0}$ leads to


$$\underset{t\to \infty }{\text{lim}}\left(x_{j}(t)-x_{j}\left(t-T_{ij}(t)\right)\right)=\text{0}$$

Then, $\underset{t\to \infty }{\text{lim}}u_{i}(t)=\text{0}$ implies that $\underset{t\to \infty }{\text{lim}}\sum_{N_{i}}a_{ij}\left(x_{i}-x_{j}\right)=\text{0}$, and subsequently $\underset{t\to \infty }{\text{lim}}Lx(t)=\text{0}$ and $\underset{t\to \infty }{\text{lim}}\left(x_{i}(t)-x_{j}(t)\right)=\underset{t\to \infty }{\text{lim}}\left(q_{i}(t)-q_{j}(t)\right)=\text{0}$ ∀i,j∈V.

Next, consider the time instants where ${\dot{v}}_{i}$ is discontinuous, i.e., ${\dot{T}}_{ij}(t)$ is discontinuous for some $j\in N_{i}$, Such a discontinuity happens when a new sampled state from a neighbor arrives at the i’th virtual system, and the set of these instants has a Lebesgue measure of zero. Consider such an instant $t_{D}$ when ${\dot{v}}_{i}$ is discontinuous, that $T_{ij}\left(t_{D}\right)$ is discontinuous for some $j\in N_{i}$. In this case, ${\dot{v}}_{i}\left(t_{D}\right)$ is discontinuous. With the fact that ${\dot{v}}_{i}\in L_{\infty }$, we have $v_{i}$ is uniformly continuous at $t_{D}$ and $x_{i}$ is differentiable at $t_{D}$. Combine the differentiability of x and the consensus result $\underset{t\to \infty }{\text{lim}}\left(x_{i}(t)-x_{j}(t)\right)$ proved on intervals where ${\dot{v}}_{i}$ is continuous, we have $\underset{t_{D}\to \infty }{\text{lim}}\left(x_{i}\left(\;t_{D}^{+}\right)-x_{j}\left(\;t_{D}^{+}\right)\right)=\underset{t_{D}\to \infty }{\text{lim}}\left(x_{i}\left(\;t_{D}^{-}\right)-x_{j}\left(\;t_{D}^{-}\right)\right)=\underset{t_{D}\to \infty }{\text{lim}}\left(x_{i}\left(\;t_{D}\right)-x_{j}\left(\;t_{D}\right)\right)=\text{0}$. Invoking the same arguments, since ${\ddot{q}}_{i}\in L_{\infty }$, $q_{i}$ is differentiable, and it can be established that $\underset{t\to \infty }{\text{lim}}\left(q_{i}\left(t_{D}\right)-q_{j}\left(t_{D}\right)\right)=\text{0}\;\forall i,j\in V$, which means the interconnected EL systems (1) reach consensus when t→∞. ◻

## 4. Numerical examples

This section provides the simulation with ten distributed 2-DoF robotic manipulators connected by sampled-data communication with time-varying delays.

The EL systems are 2-DOF manipulators with $M_{i}\left(q_{i}\right)=[\begin{array}{cc} m_{\text{11}i} & m_{\text{12}i}\\ m_{\text{21}i} & m_{\text{22}i} \end{array}]$, $C_{i}\left(q_{i},{\dot{q}}_{i}\right)=[\begin{array}{cc} c_{\text{11}i} & c_{\text{12}i}\\ c_{\text{21}i} & \text{0} \end{array}]$ and $g_{i}\left(q_{i}\right)=[\begin{array}{c} g_{\text{1}i}\\ g_{\text{2}i} \end{array}]$, where

$m_{\text{11}i}=m_{\text{1}i}l_{c\text{1}i}^{\text{2}}+m_{\text{2}i}\left(l_{\text{1}i}^{\text{2}}+l_{c\text{2}i}^{\text{2}}+\text{2}l_{\text{1}i}l_{c\text{2}i}\;\text{cos}\left(q_{\text{2}i}\right)\right)+J_{\text{1}}+J_{\text{2}}$,

$m_{\text{12}i}=m_{\text{2}i}\left(l_{c\text{2}i}^{\text{2}}+l_{\text{1}i}l_{c\text{2}i}\;\text{cos}\left(q_{\text{2}i}\right)\right)+J_{\text{2}}$,

$m_{\text{22}i}=m_{\text{2}i}l_{c\text{2}i}^{\text{2}}+J_{\text{2}}$,

$c_{\text{11}i}=-m_{\text{2}i}l_{\text{1}i}l_{c\text{2}i}\;\text{sin}\left(q_{\text{2}i}\right){\dot{q}}_{\text{2}i}$,

$c_{\text{12}i}=-m_{\text{2}i}l_{\text{1}i}l_{c\text{2}i}\;\text{sin}\left(q_{\text{2}i}\mathrm{\backslash rightleft}({\dot{q}}_{\text{1}i}+{\dot{q}}_{\text{2}i}\right)$,

$c_{\text{21}i}=m_{\text{2}i}l_{\text{1}i}l_{c\text{2}i}\;\text{sin}\left(q_{\text{2}i}\right){\dot{q}}_{\text{1}i}$,

$g_{\text{1}i}=\left(m_{\text{1}i}l_{c\text{1}i}+m_{\text{2}i}\right)g\;\text{sin}\left(q_{\text{1}i}\right)+m_{\text{2}i}l_{c\text{2}i}g\;\text{sin}\left(q_{\text{1}i}+q_{\text{2}i}\right)$,

$g_{\text{2}i}=m_{\text{2}i}l_{c\text{2}i}g\;\text{sin}\left(q_{\text{1}i}+q_{\text{2}i}\right)$.

The parameters are given by $J_{\text{1}}=\text{2}$, $J_{\text{2}}=\text{0}.\text{5}$;

For agents 1 and 2, $m_{\text{1}i}=\text{3}$, $m_{\text{2}i}=\text{2}$, $l_{\text{1}i}=\text{0}.\text{5}$, $l_{c\text{1}i}=\text{0}.\text{4}$, $l_{c\text{2}i}=\text{0}.\text{5}$.

For agents 3 and 4, $m_{\text{1}i}=\text{4}$, $m_{\text{2}i}=\text{3}$, $l_{\text{1}i}=\text{0}.\text{4}$, $l_{c\text{1}i}=\text{0}.\text{5}$, $l_{c\text{2}i}=\text{0}.\text{4}$.

For the remaining six agents, $m_{\text{1}i}=\text{2}$, $m_{\text{2}i}=\text{4}$, $l_{\text{1}i}=\text{0}.\text{3}$, $l_{c\text{1}i}=\text{0}.\text{5}$, $l_{c\text{2}i}=\text{0}.\text{5}$.

The communication topology has the following graph Laplacian L


$$L=[\begin{array}{cccccccccc} \text{1}\text{4} & \text{0} & -\text{3} & \text{0} & \text{0} & \text{0} & \text{0} & -\text{4} & \text{0} & -\text{7}\\ \text{0} & \text{9} & \text{0} & -\text{8} & \text{0} & \text{0} & \text{0} & \text{0} & \text{0} & -\text{1}\\ -\text{3} & \text{0} & \text{5} & \text{0} & \text{0} & \text{0} & \text{0} & -\text{2} & \text{0} & \text{0}\\ \text{0} & -\text{8} & \text{0} & \text{1}\text{0} & \text{0} & \text{0} & \text{0} & \text{0} & -\text{2} & \text{0}\\ \text{0} & \text{0} & \text{0} & \text{0} & \text{8} & \text{0} & -\text{5} & \text{0} & -\text{3} & \text{0}\\ \text{0} & \text{0} & \text{0} & \text{0} & \text{0} & \text{6} & \text{0} & -\text{4} & -\text{2} & \text{0}\\ \text{0} & \text{0} & \text{0} & \text{0} & -\text{5} & \text{0} & \text{1}\text{4} & \text{0} & \text{0} & -\text{9}\\ -\text{4} & \text{0} & -\text{2} & \text{0} & \text{0} & -\text{4} & \text{0} & \text{1}\text{0} & \text{0} & \text{0}\\ \text{0} & \text{0} & \text{0} & -\text{2} & -\text{3} & -\text{2} & \text{0} & \text{0} & \text{7} & \text{0}\\ -\text{7} & -\text{1} & \text{0} & \text{0} & \text{0} & \text{0} & -\text{9} & \text{0} & \text{0} & \text{1}\text{7} \end{array}]$$

**Fig 1:**
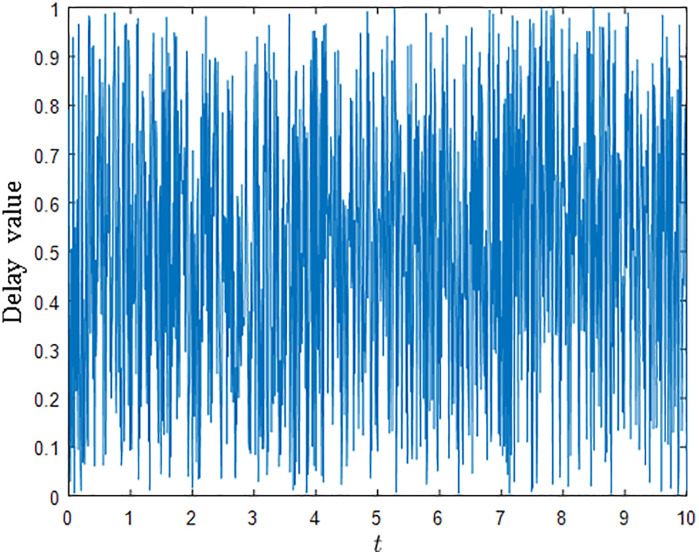
The random communication delay.

The communication delays are different on each channel and randomly changing every 0.01 second. Fig 1 shows an example of the random delay during a period of 10 seconds. The maximum value of the delays is T=1. The sampling interval for the agents are chosen as h=1. Condition (16) gives $d_{i}>(T+h)\Delta_{i}$ where T+h=2 and the maximum $\Delta_{i}$ is 17. So the damping gains are chosen as $d_{i}=\text{4}\text{0}$ for all agents to satisfy the condition (16). The coefficient $k_{i}$ are chosen to be 20 for all agents. The initial values of the virtual systems are $x_{i}(\text{0})=\text{0}$ and $v_{i}(\text{0})=\text{0}$ for all agents.

**Fig 2:**
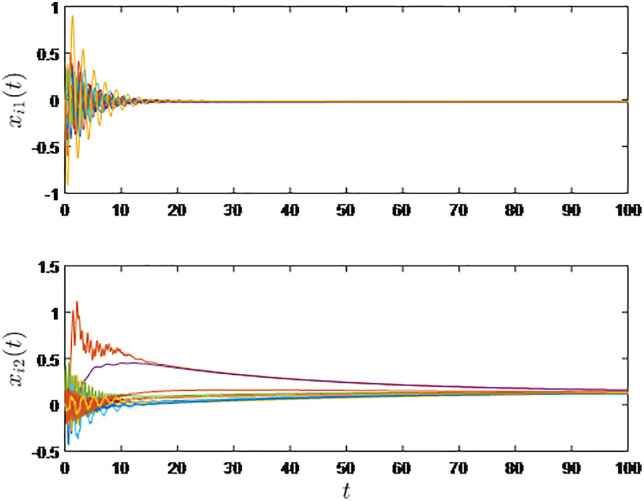
Virtual positions of the ten virtual systems associated with the EL systems.

**Fig 3:**
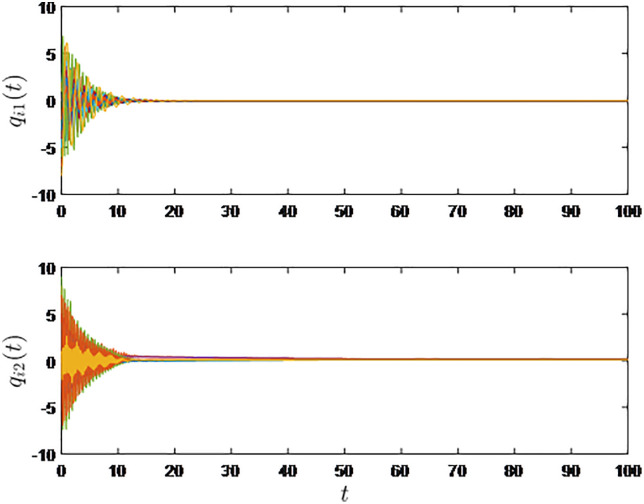
Generalized positions of the ten robotic manipulators.

It is shown in Fig 2 and Fig 3 that consensus is obtained for the sampled-data EL systems under the influece of the random time-varying communication delay.

## 5. Conclusion

This paper presented a solution to the sampled-data consensus problem for networked Euler-Lagrange systems under irregular communication delays and without access to velocity measurements. By leveraging a passivity-based control (PBC) framework with virtual systems and in-depth analysis, our approach removes the impractical requirement of finite delay derivatives, a limitation in previous studies. We have established a definitive condition for the controller damping gains required to achieve consensus. The proposed algorithm’s effectiveness and ability to handle discontinuous delays and thus sampled-data communication were validated through numerical simulations. This work extends the applicability of PBC methods to more realistic and challenging scenarios in networked control systems.
